# Boots or No Boots: The Advantages of Clogs

**Published:** 1918-10-19

**Authors:** R. King Brown

**Affiliations:** Medical Officer of Health. Public Health Department, Town Hall, Bermondsey, S.E. 16.


*To the Editor of* The Hospital.

### Boots or No Boots: The Advantages of Clogs.

*To the Editor of* The Hospital.

Sir,—On the question of boots for school children, I saw the suggestion made in one of the weekly Sunday papers that clogs might be substituted in place of the ordinary leather boots. I had in my mind a suggestion of this sort when I wrote to you previously, and it is well worth consideration. There is only one objection that I know of against clogs, and that is the noise; but this can be obviated if the soles are made of sufficiently hard wood that it does not necessitate them being shod with iron. Anybody who has worn clogs knows that they keep the feet much warmer than ordinary boots. They last longer, are water-tight, and are much cheaper in every way than boots. As several writers have pointed out, the present cheap boots are perfect rubbish, as the soles are mostly made up with cardboard. A shower of rain renders them damp inside, and I am quite certain that damp feet, especially in growing children, sow the seeds of disease, not only immediately, but in after life.

During the coming winter we shall all suffer more or less from shortage of coal, and it is therefore doubly important that the matter of footwear should be taken in hand. Most people can supply clogs for themselves, but I think the suggestion of the writer in *Reynolds's* that a certain number should be given free, the same as dinners, is a good one, and I trust the matter will be taken up at an early date by the education authorities.—Your obedient servant,

R. King Brown,
Medical Officer of Health.

Public Health Department, Town Hall,
Bermondsey, S.E. 16., October 12, 1918.

